# Supplementary figures and images for: SNX10 functions as a modulator of piecemeal mitophagy and mitochondrial bioenergetics
Source: J Cell Biol. 2025 Mar 7;224(5):e202404009. doi: 10.1083/jcb.202404009 (PMC11893173; doi:10.1083/jcb.202404009)

Source Data Fig. 1

C

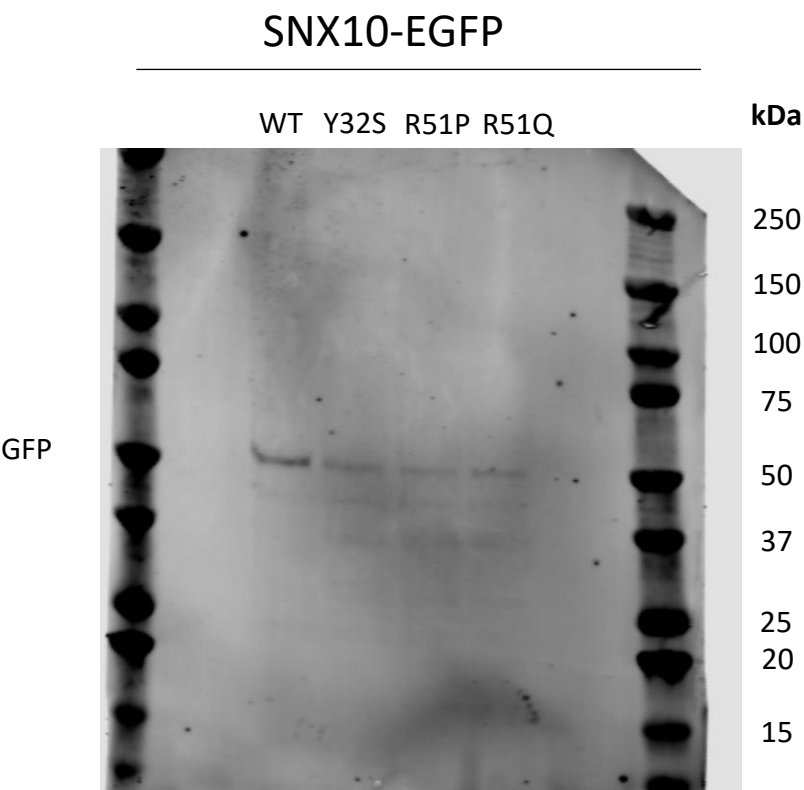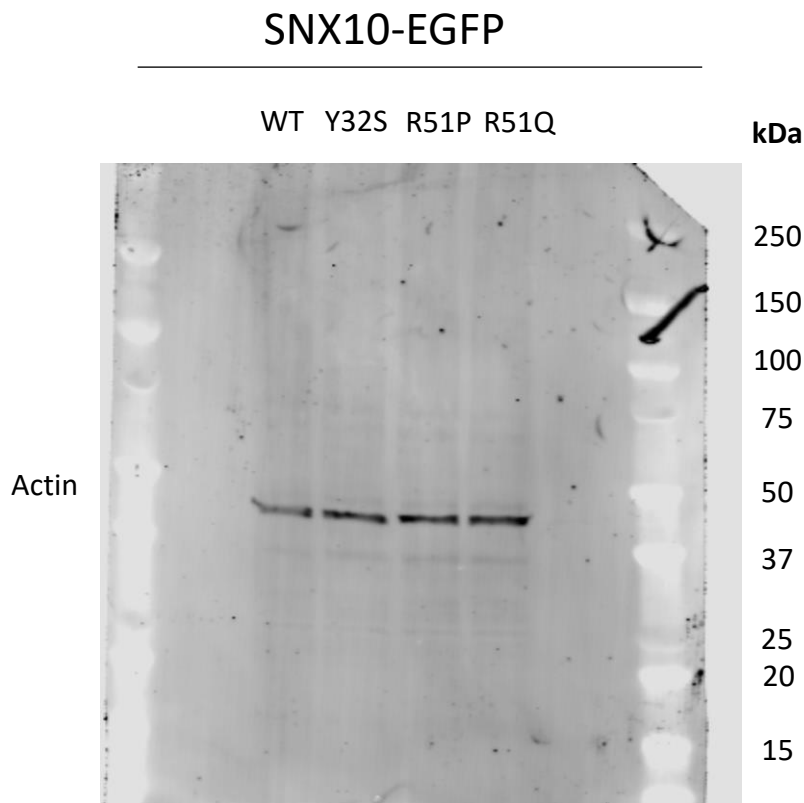

Supplement: SourceData F1 — is the source file for Fig. 1. [file jcb_202404009_sourcedataf1.pdf]

Source Data Fig. 2

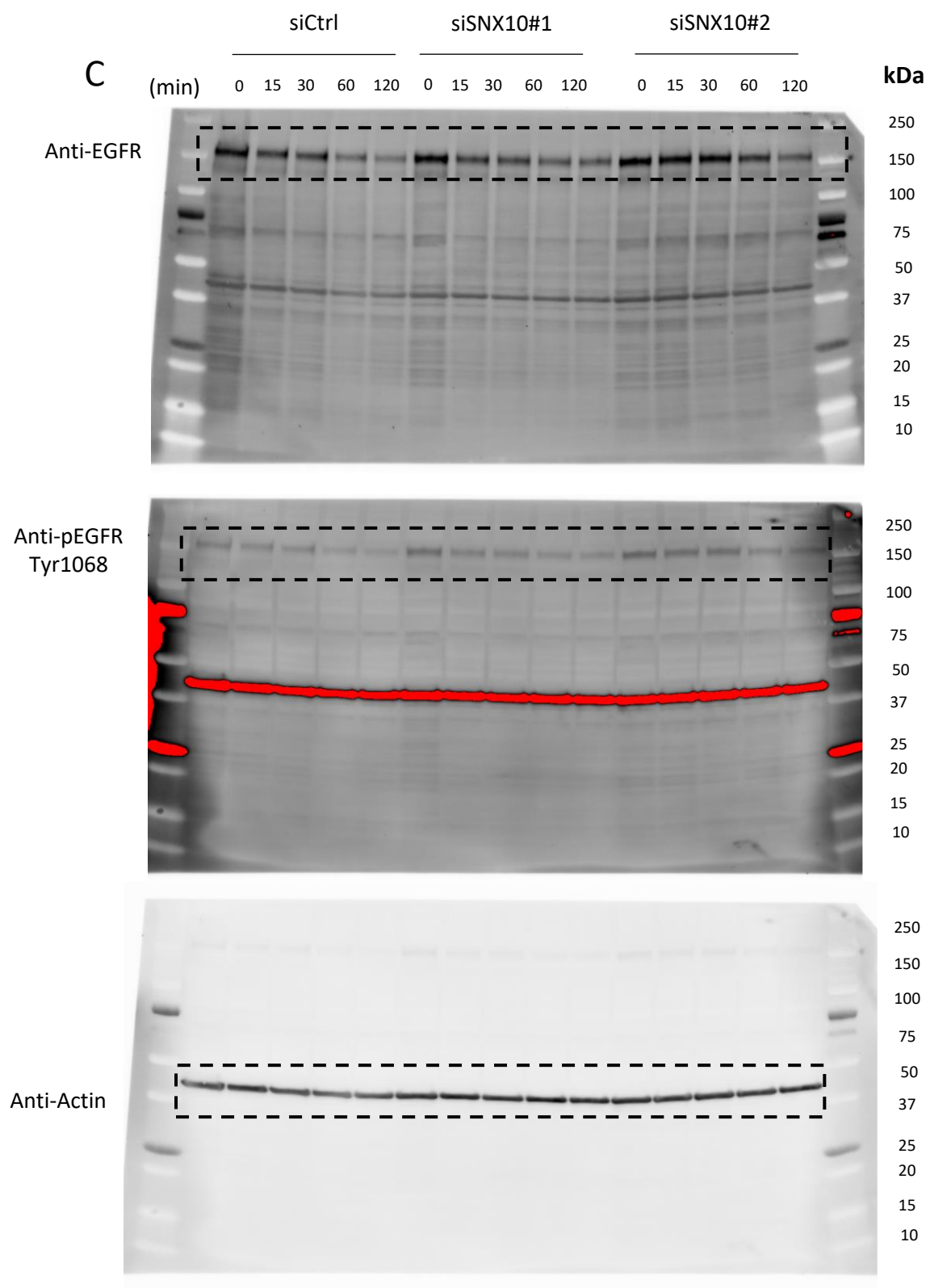

Supplement: SourceData F2 — is the source file for Fig. 2. [file jcb_202404009_sourcedataf2.pdf]

Source Data Fig. 6

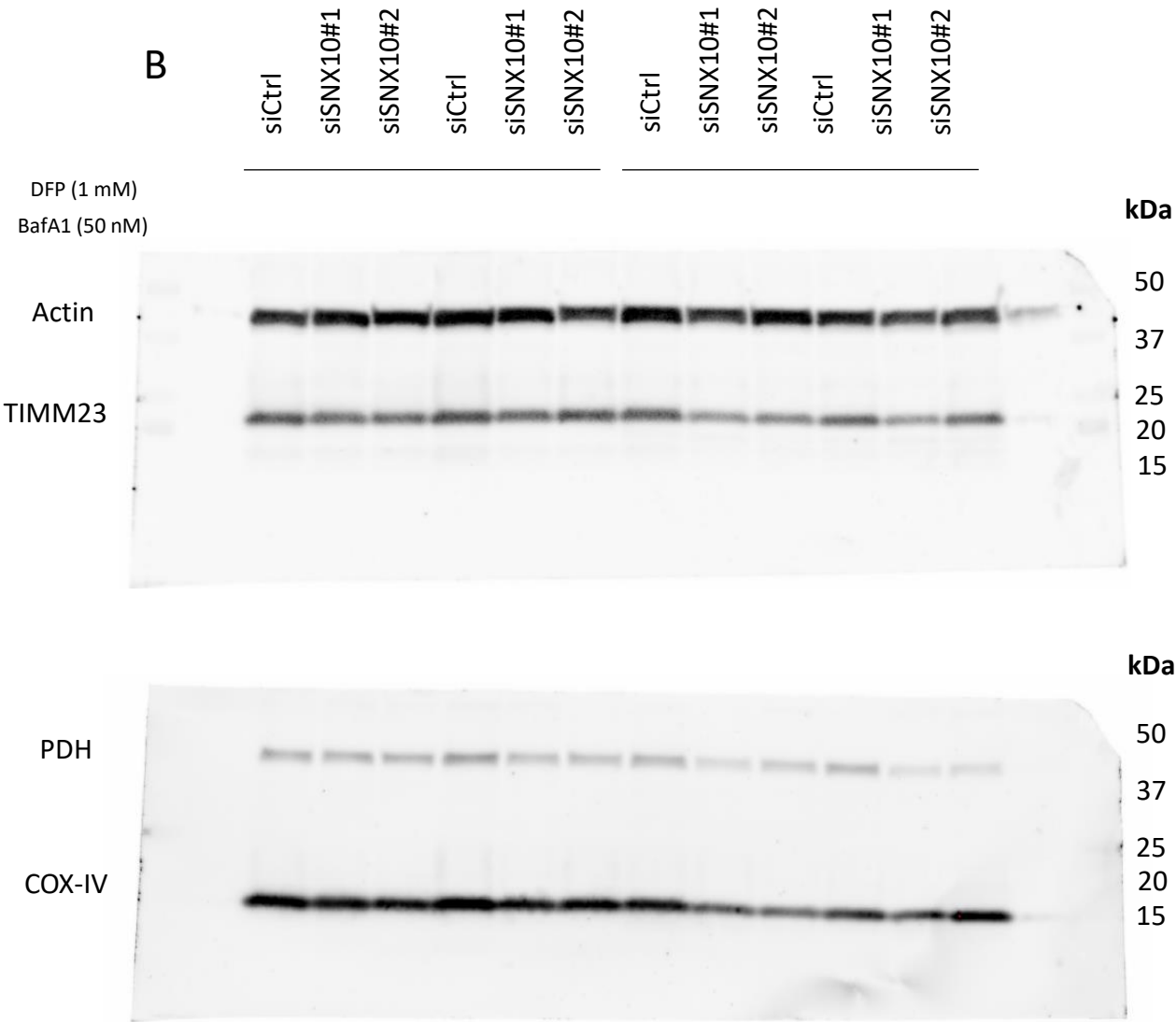

Supplement: SourceData F6 — is the source file for Fig. 6. [file jcb_202404009_sourcedataf6.pdf]

Source Data Fig. 7

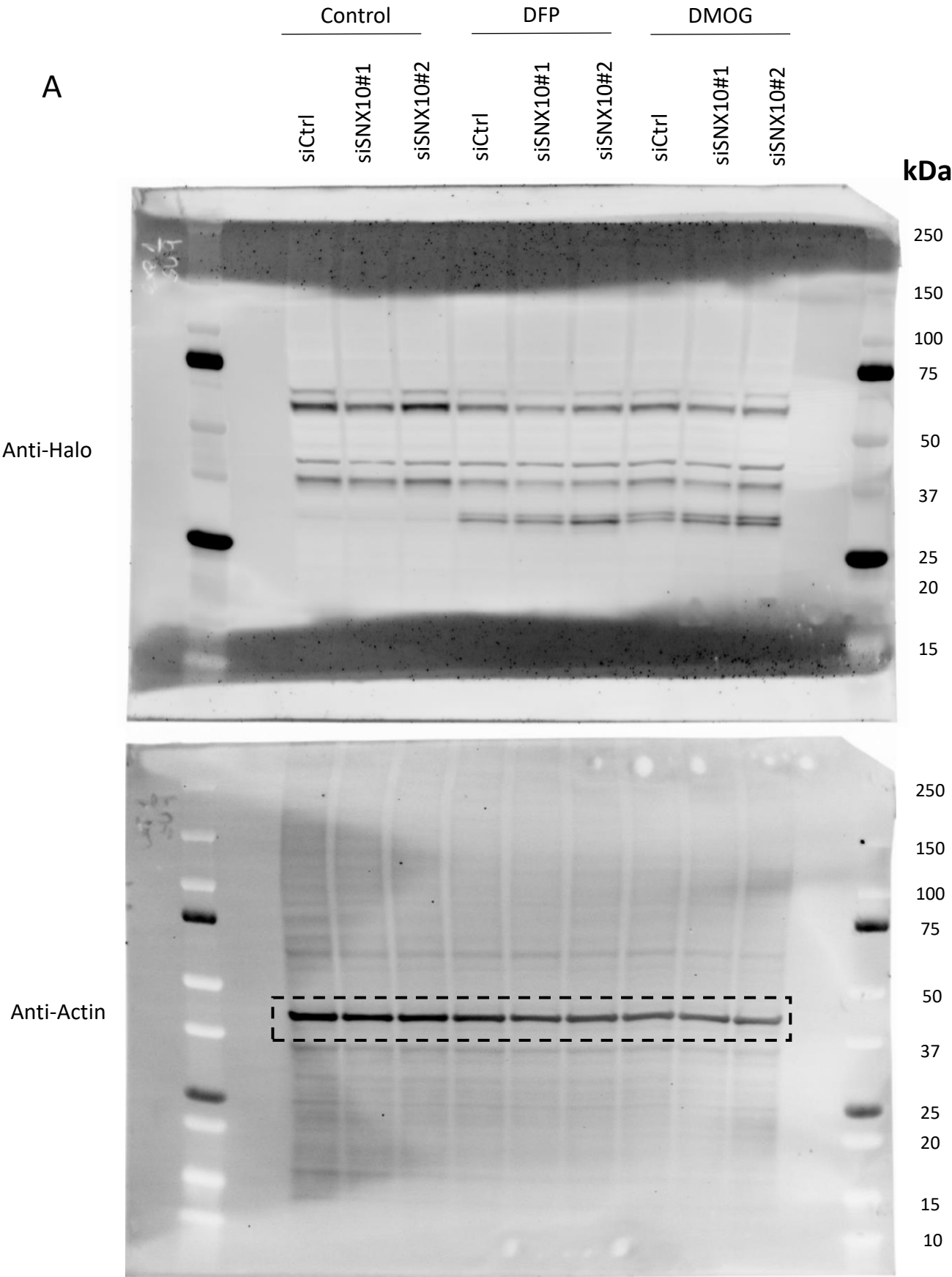

Supplement: SourceData F7 — is the source file for Fig. 7. [file jcb_202404009_sourcedataf7.pdf]

## Source Data Fig. 8

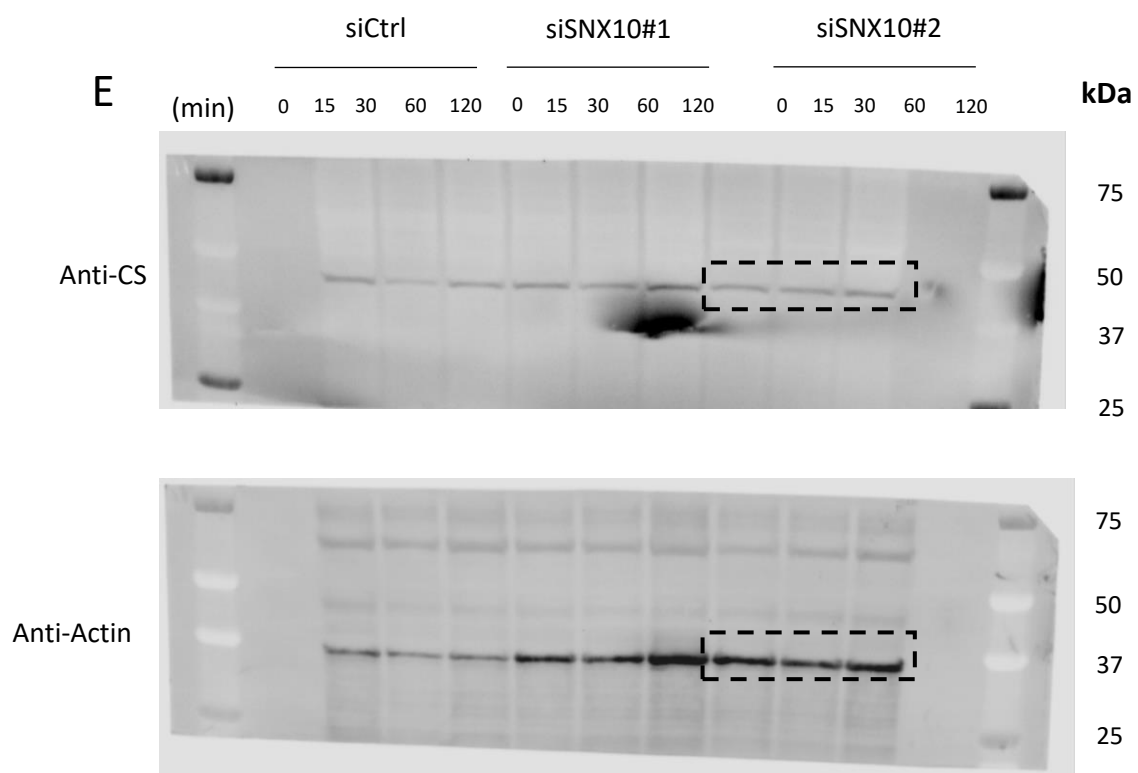

Supplement: SourceData F8 — is the source file for Fig. 8. [file jcb_202404009_sourcedataf8.pdf]

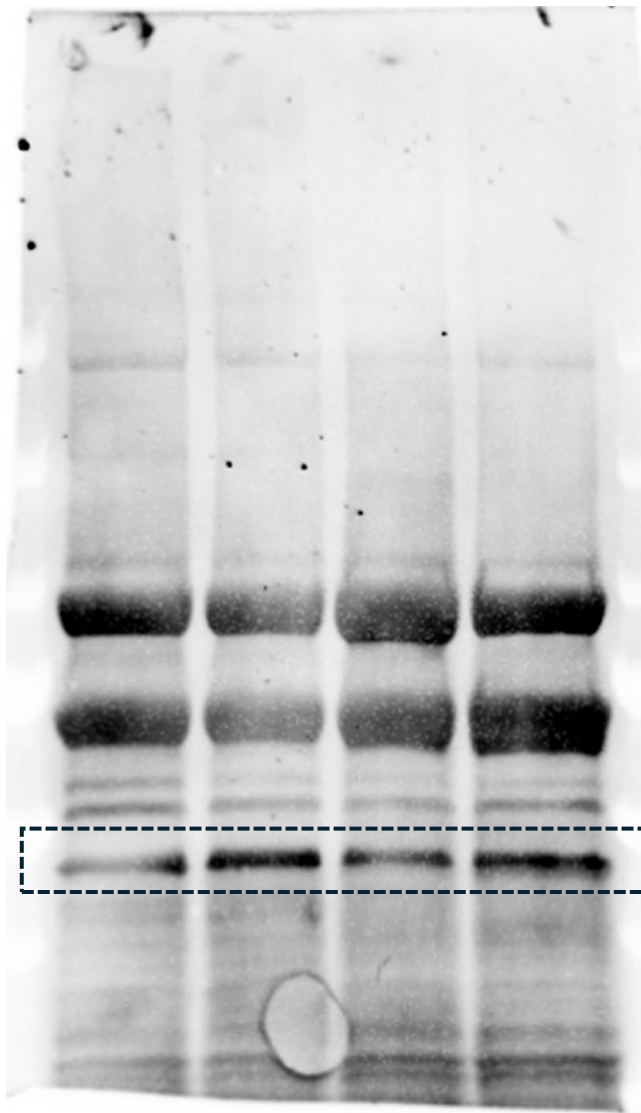

Anti-actin

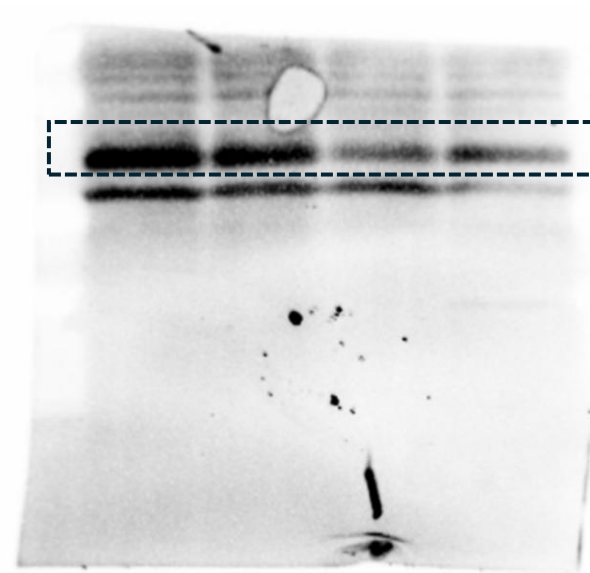

Anti-cox IV

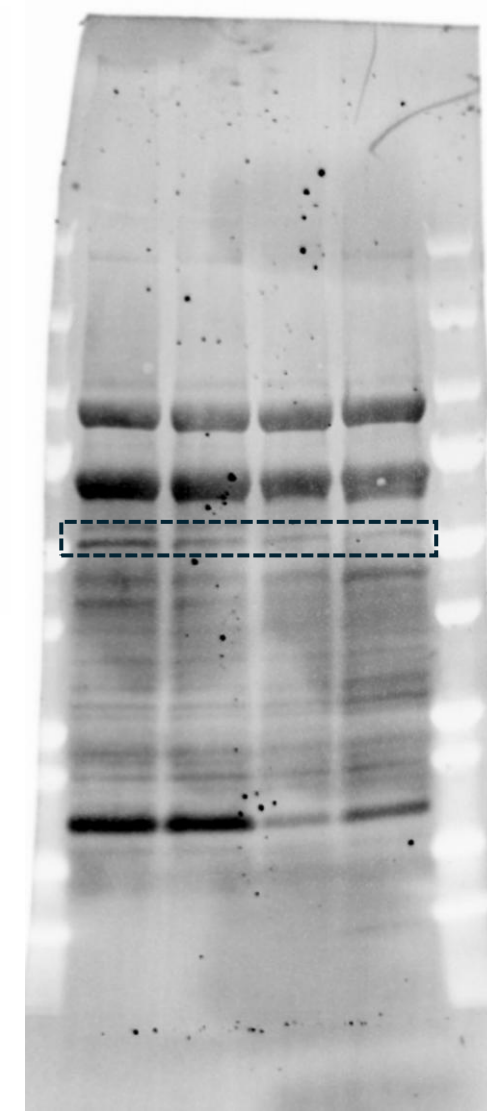

Anti-samm50

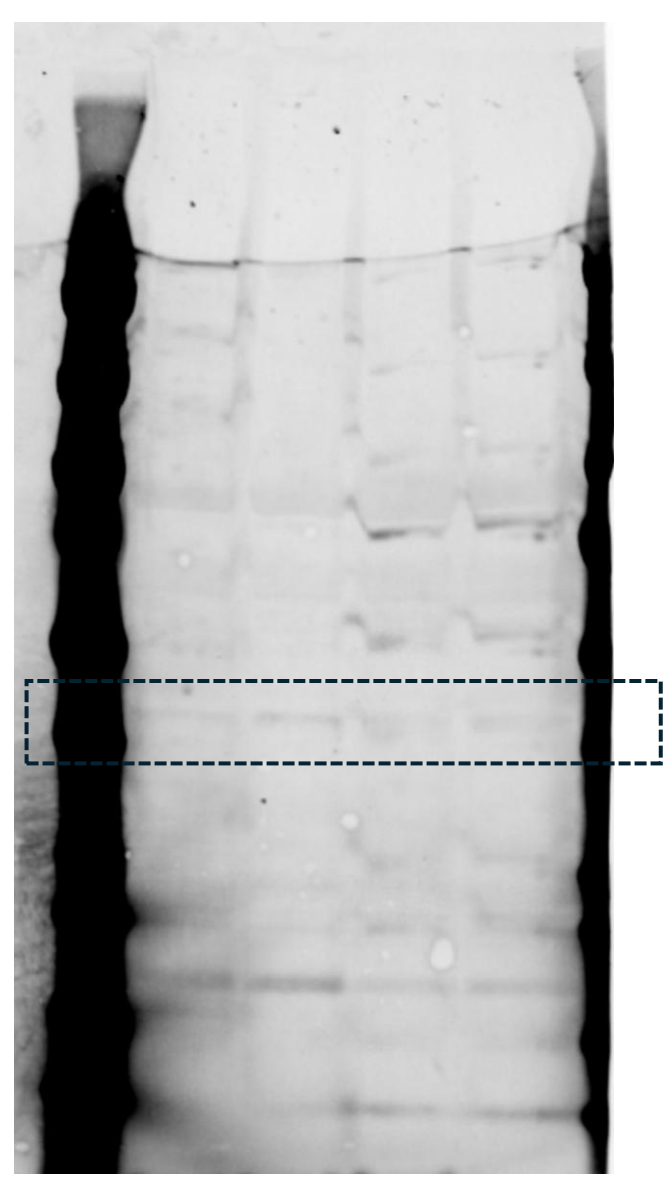

Anti-snx10

Supplement: SourceData F9 — is the source file for Fig. 9. [file jcb_202404009_sourcedataf9.pdf]
